# The Southern Surgical and Gynecological Association

**Published:** 1898-11

**Authors:** 


### The Southern Surgical and Gynecological Association.

The eleventh annual meeting of the Association which was announced to be held in Memphis, Tenn., Tuesday, Wednesday and Thursday, November 8th, 9th and 10th, has been postponed till Tuesday, Wednesday and Thursday, December 6th, 7th and 8th, 1898, on account of the quarantine regulations in some parts of the South. The Gayoso House has been selected as headquarters for the Association.

The following is a partial list of the papers to be read:

1. President's Address, Richard Douglas, M. D., Nashville, Tenn.
2. Gunshot wounds, W. E. Parker, M. D., New Orleans, La.

3. Electro therapeutics in medicine and surgery, Jas. McF. Gaston, Atlanta, Ga.

4. The normal position of the uterus defined, A. H. Buckmaster, M. D., Charlottesville, Va.

5. Abdominal opening for intra-peritoneal surgical work, Jos. Price, M. D., Philadelphia, Pa.

6. The choice of material for ligatures and sutures in gynecological surgery, L. S. McMurtry, M. D., Louisville, Ky.

7. Repair in cases of complete tear of the perineum, Howard A. Kelly, M. D., Baltimore, Md.

8. Conservative treatment of the diseased ovary, Jos. Taber Johnson, M. D., Washington, D. C.

9. Thoracotomy for tumors involving the ribs, F. W. Parham, M. D., New Orleans, La.

10. The use and abuse of normal salt solution, J. W. Bovee, M. D., Washington, D. C.

11. A report of fifty prostatectomies, with remarks on the treatment of prostatic overgrowth in the aged, John T. Bryson, M. D., St. Louis, Mo.

12. Remarks on the surgery of the gall-bladder and bile-ducts, A. V. L. Brokaw, M. D., St. Louis, Mo.

13. Past and present surgery of the gall-bladder and bile-ducts, Wm. H. Myers, M. D., Fort Wayne, Ind.

14. The pelvic floor, its functions, injuries and repair, M. C. McGannon, M. D., Nashville, Tenn.

15. When should we operate for appendicitis? A. M. Cartledge, M. D., Louisville, Ky.

16. Ureteral anastomosis, Geo. H. Noble, M. D., Atlanta, Ga.

17. Ovarian cysts as a complication of pregnancy and labor, J. W. Long, M. D., Salisbury, N. C.

18. Incised wounds of the larynx, Edwin Walker, M. D., Evansville, Ind.

19. Tubal pregnancy; primary rupture into the broad ligament and secondary into the peritoneum; laparotomy, convalescence complicated by septic diarrhœa and metastatic abscess of the liver, R. Matas, M. D., New Orleans, La.

20. Removal of partially descended, infected, strangulated testicle, complicated by hernia, R. R. Kime, M. D., Atlanta, Ga.

21. The diagnosis of tubercular peritonitis and indications for surgical treatment, W. L. Robinson, M. D., Danville, Va.

22. Foreign bodies in the œsophagus with report of cases, A. Vander Veer, M. D., Albany, N. Y.

23. Penetrating wounds of the abdomen, Floyd W. McRae, M. D., Atlanta, Ga.

24. The management of pregnancy complicating intra-abdominal tumors, with cases, Rufus B. Hall, M. D., Cincinnati, O.

25. The rarity of ovarian tumors in negresses, I. S. Stone, M. D., Washington, D. C.

26. Tumors of the breast, W. F. Westmoreland, M. D., Atlanta, Ga.

27. Penetrating wounds of the chest, J. B. Murfree, Murfreesboro, Tenn.

28. Surgery of the pelvic organs without speculums or retractors, W. H. Wathen, M. D., Louisville, Ky.

29. Report of a case of splenectomy for wandering hypertrophied spleen, Wyatt Heflin, M. D., Birmingham, Ala.

30. Cœliotomy in the treatment of retroverted pregnant uterus when incarcerated, Henry D. Fry, M. D., Washington, D. C.

31. Odds and ends in pelvic surgery, Walter B. Dorsett, M. D., St. Louis, Mo.

32. Treatment of pelvic inflammation, Jas. A. Goggans, Alexander City, Ala.

33. Mechanical aids in intestinal surgery, J. D. S. Davis, M. D., Birmingham, Ala.

34. The history of myomectomy, Chas. P. Noble, M. D., Philadelphia, Pa.

35. Observations upon cranial operations with report of cases, Wm. Perrin Nicholson, M. D., Atlanta, Ga.

36. Plastic surgery in gynecology, W. D. Haggard, Jr., Nashville, Tenn.

37. Ventro-fixation for retro-displacements of the uterus, R. J. Trippe, M. D., Chattanooga, Tenn.

38. Removal of five gallon ovarian cyst from girl seventeen years old, R. R. Kime, M. D., Atlanta, Ga.

39. Transpleural hepatotomy by resection of the rib and free incision; recovery, R. Matas, M. D., New Orleans, La.

40. Subject to be announced, W. S. Elkins, M. D., Atlanta, Ga.

41. Surgery of the stomach, W. E. B. Davis, M. D., Birmingham, Ala.

Members of the medical profession are cordially invited to attend. Dr. R. B. Maury, of Memphis, is chairman of the committee of arrangements.

RICHARD DOUGLAS, M. D., President.

W. E. B. DAVIS, M. D., Secretary.

